# Infectious Discitis and Spondylodiscitis in Children

**DOI:** 10.3390/ijms17040539

**Published:** 2016-04-09

**Authors:** Nicola Principi, Susanna Esposito

**Affiliations:** Pediatric Highly Intensive Care Unit, Department of Pathophysiology and Transplantation, Università degli Studi di Milano, Fondazione IRCCS Ca’ Granda Ospedale Maggiore Policlinico, 20122 Milan, Italy; nicola.principi@unimi.it

**Keywords:** discitis, osteoarticular infections, pediatric orthopedics, spondylodiscitis

## Abstract

In children, infectious discitis (D) and infectious spondylodiscitis (SD) are rare diseases that can cause significant clinical problems, including spinal deformities and segmental instabilities. Moreover, when the infection spreads into the spinal channel, D and SD can cause devastating neurologic complications. Early diagnosis and treatment may reduce these risks. The main aim of this paper is to discuss recent concepts regarding the epidemiology, microbiology, clinical presentation, diagnosis, and treatment of pediatric D and SD. It is highlighted that particular attention must be paid to the identification of the causative infectious agent and its sensitivity to antibiotics, remembering that traditional culture frequently leads to negative results and modern molecular methods can significantly increase the detection rate. Several different bacterial pathogens can cause D and SD, and, in some cases, particularly those due to *Staphylococcus aureus*, *Kingella kingae*, *Mycobacterium tuberculosis*, *Brucella* spp., the appropriate choice of drug is critical to achieve cure.

## 1. Introduction

In children, infectious discitis (D), *i.e.*, infection of the vertebral disc, and infectious spondylodiscitis (SD), *i.e.*, the simultaneous infection of a vertebral disc and the adjacent vertebral bodies, are rare diseases that can cause significant clinical problems [1,2,3,4,5,6,7]. Childhood SD is a term frequently used for encompassing a continuum of spinal infections, from D to vertebral osteomyelitis through SD. D and SD are usually divided into pyogenic (*i.e*., the most frequent); unspecific granulomatous; specific (such as tuberculosis); and parasitic. In industrialized countries, pyogenic SD is the most frequent form and other presentations are exceptional. In the time before the use of antibiotics, mortality due to pyogenic D and SD reached 90% [8]. Currently, due to better diagnostic tools and improvements in medical and surgical treatment, mortality is significantly reduced and is lower than 5%. However, in a not insignificant number of cases, D and SD can lead to serious spinal deformities and segmental instabilities. Finally, when the infection spreads into the spinal channel, D and SD can cause devastating neurologic complications. These severe complications are quite common in non-pyogenic infections, including cases due to *Mycobacterium tuberculosis* that are endemic in some developing and emerging countries but are diagnosed also in the industrialized world. Early diagnosis and treatment might reduce these risks. However, unfortunately, the early diagnosis of D and SD in children, particularly in young children, is very difficult. Moreover, identification of the causative infectious agents is frequently impossible, imaging is poorly contributive in the first period of the disease’s development, and the best therapeutic approach is not precisely defined. Consequently, subacute and chronic cases can frequently have a negative outcome. The main aim of this paper is to discuss recent concepts regarding pediatric D and SD. The literature search was performed using PubMed and considered all of the papers published in the English language since 1 January 2010, using the key words: “discitis” or “spondylodiscitis” and “children” or ”pediatrics” or “pediatric age.” Clinicians caring for children must be especially well versed in these concepts as they can be used to guide evaluation and treatment, thereby reducing the risk of major spinal complications following these diseases.

## 2. Epidemiology

Infectious D and SD have been diagnosed with increasing frequency [9,10]. However, D and SD remain uncommon entities among pediatric patients.

In the period in which MRI was not available, the incidence of SD was estimated by Digby and Kersley to be approximately 1:250,000 of the population [1]. Some years later, Cushing calculated that cases primarily involving the disc were 1–2 per year per 32,500 pediatric hospital evaluations [2]. Cases that began with vertebral involvement account for 1%–2% of all the cases of pediatric osteomyelitis. A more recent evaluation carried out in a French pediatric orthopedic unit where more modern methods for diagnosis were in use has estimated that D and SD together accounted for approximately 3% of all the cases of osteoarticular infections (OAIs) admitted to the unit [11]. However, it must be highlighted that all of these data are from industrialized countries, where the diagnosis and treatment of infectious diseases is significantly more effective than in the developing world. It is likely in these geographic areas that the incidence of D and SD is even higher because cases due to tuberculosis are probably more common. Approximately 5% of all the cases of pediatric extrapulmonary tuberculosis involve joints and bones, and vertebral lesions are probably the most common involvement [12].

Some studies have highlighted a triphasic age distribution of D and SD in children [1,2,3,4,5,6,7]. The first peak incidence is in children aged only a few weeks or months old, the second in those between 6 months and the end of the preschool period, and the third in school-aged children.

## 3. Pathogenesis of Discitis and Spondylodiscitis

In most patients, pathogens reach the spine hematogenously, starting from a previously existing site of infection [2,3]. However, exceptionally, pathogens can be directly inoculated from a diagnostic or surgical procedure or following a trauma. Interestingly, in infants, D and SD can derive from unwitnessed button battery ingestion. Several cases with this origin have been described [13,14,15].

When bacteria reach the spine starting from a distant focus, the infection can first involve the disc and subsequently reach the adjacent vertebral endplates and the entire vertebral bodies or originate in the vertebral bone tissue as vertebral osteomyelitis, subsequently involving the disc [16,17]. The first possibility is significantly more common in younger children, whereas the second is characteristic of older children and adolescents. The peculiar vascularization of the vertebral disc and the vertebral body during development explains this difference. Although no blood or lymph vessels are present in the nucleus pulposus at any age, supply vessels persist in the cartilaginous vertebral endplate until the seventh year of life, and those in the annulus fibrosus can persist up to the age of 20 years [16,17]. This persistence enables bacterial emboli to be deposited within the disc itself, with possible extension of the infection to vertebral endplates and vertebral bodies and, rarely, to the paravertebral area and the epidural space. In older children and adolescents, the subchondral spongy bone is supplied by end arteries where a small septic embolus may lodge in the setting of the bacteremia and begin to proliferate, leading to bone infarction and subsequent vertebral osteomyelitis. From here, the infection can spread by direct extension with rupture of the infective focus through the endplate into the disc.

However, most authors currently believe that pure D does not exist for the already mentioned vascular anatomical reasons. These authors consider that all bacterial infections are primarily located in the metaphyseal region of the vertebral body, and that the microorganism crosses the cartilaginous vertebral plate, runs through the surface of the disc via the anastomotic branches, infects the adjacent vertebral metaphysis, and reaches finally the disc space between the two vertebral bodies involved.

## 4. Etiology

Many attempts to identify the causative pathogen of D and SD of children through blood and/or disc/vertebral aspiration cultures fail, causing related problems in selecting the most appropriate antibiotic therapy [18,19,20,21,22]. When positive, pyogenic bacteria are usually detected, with *Staphylococcus aureus* being the cause of D and SD in approximately 80% of the cases that occur in the first months of life and in most of those that develop in older children [1,2,3,4,5,6,7]. Other agents less frequently identified are coagulase-negative *Staphylococcus*, α-hemolytic *Streptococcus*, *Streptococcus pneumoniae*, and Gram-negative rods such as *Escherichia coli* and *Salmonella* spp. In children between 6 months and 4 years of age, a group in whom traditional culture is very frequently negative, recent studies seem to indicate that a relevant etiologic role might be played by *Kingella kingae*. *K. kingae* is a Gram-negative organism that is difficult to detect in different body fluids [23]. However, using aerobic blood culture vials or real-time polymerase chain reaction (PCR), the bacterium can be more easily identified. The use of molecular methods has led to the identification of this pathogen as the cause of many OAIs that were previously considered of unknown origin because of negative cultures [24]. Currently, *K. kingae* is considered the leading cause of bacterial OAIs in children aged less than 4 years, excluding the neonatal period. Moreover, it was evidenced that in a great number of children with OAIs and negative culture, throat swabs were positive for *K. kingae*, suggesting that a throat swab could provide strong evidence that the microorganism was responsible for the OAI, or even stronger evidence that it was not [25]. Ceroni *et al.* reported that in a number of pediatric SD cases, *K. kingae* DNA could be found in the oropharynx, providing reasonable suspicion that this microorganism was responsible for the diseases [26]. However, in two out of 10 cases the pathogen was also detected in the blood of the affected children. Moreover, further support for the hypothesis that *K. kingae* might be an important causative agent of D and SD in children 6 months to 4 years old is given by the evidence that *K. kingae* has limited virulence, and most of the cases occurring in children of this age range spontaneously have a favorable evolution [27].

Subacute and chronic D and SD can be due to a wide spectrum of non-pyogenic bacteria such as *Mycobacterium tuberculosis*, *Brucella* spp. and fungi (*i.e.*, *Aspergillus* spp., *Candida* spp. and *Cryptococcus neoformans*) [28]. Clinical history might suggest the diagnosis. Diagnostic tests, such as blood culture or antigen titers in the case of *Brucella* spp. or antigen detection for fungal infection can be useful for the correct diagnosis [4]. However, direct aspiration or tissue biopsy is required for the identification of the etiologic agent.

Table 1 summarizes the main etiologic agents for D and SD in pediatric age and their characteristics.

## 5. Clinical Manifestations

The lumbar region of the spine is the most common localization, followed by the thoracic region and the cervical region [29,30].

Clinical manifestations of D and SD can significantly vary [31,32]. Cases occurring in neonates or younger infants are usually the most severe and are frequently associated with sepsis and multiple infectious foci. The vertebrae can be severely damaged and sometimes entirely destroyed, leading to kyphosis. Tsirikos and Tome-Bermejo described an 8-week-old boy who developed severe thoracic SD following pneumonia and sepsis [33]. Complete destruction of the T4 and T5 vertebral bodies and adjacent discs with a paraspinal abscess extending into the mediastinum and epidural spaces was evidenced. Despite antibiotic treatment and aspiration of the abscess at the age of 6 months, the child had to undergo posterior spinal fusion *in situ* to stabilize the spine and prevent progressive kyphosis. At the age of 13 months, imaging showed a lack of anterior vertebral body re-growth and anterior spinal fusion from T3 to T6; augmentation of the posterior fusion was needed.

In toddlers and preschool-aged children, signs and symptoms of disease are frequently mild. Most patients have low-grade fever, and only a minority exhibit significant neurologic manifestations. Garron *et al.*, who reviewed the medical records of 42 children with a mean age of 4.6 years reported that most patients had low-grade fever (only six had temperature >38.5 °C), and only 4 cases (10%) presented with neurologic signs on admission [31]. Two had unilateral sciatic pain, one had upper limb diplegia, and one presented with flaccid paraplegia. Pain in the spine was always combined with stiffness. Similar data were reported by Kayser *et al.*, who found that all of the 25 studied patients presented with uncharacteristic signs and symptoms [4]. None had neurologic deficits. All of the children over 1 year of age complained of back aches. Reduced general conditions were evidenced in 7 (28%) of the cases; refusal, reduced ability, or inability to walk or sit were seen in 6 (24%); and/or the need for support when standing up in 5 (20%). The child with cervical involvement had stiffness of the cervical spine and neck and shoulder pain.

Because early clinical manifestations are nonspecific, the diagnosis of D and SD frequently cannot be established until relatively late in the course of the disease. Delays of 4–6 months have been described by several authors [2,33,34]. Finally, older children and adolescents are more prone to developing vertebral osteomyelitis [3,4] and are febrile and ill-appearing, with a clinical picture resembling that evidenced in the elderly [35,36].

Non-pyogenic SD have a subacute or chronic course. In vertebral tuberculosis, it is possible to observe SD with the destruction of ≥two contiguous vertebrae and concomitant disc infection, and spondylitis in absence of disc infection [37]. The clinical pictures are suggestive of systemic illness with the possibility of serious complications. Bone loss and disturbed growth potential are common. Moreover, the growth potential is disturbed when the disease focus is surgically intervened. Cervical lesions are characterized by torcicollis, neck problems, or dysphagia. The worst complications are para- or tetraplegia [38,39]. Edema of the spinal cord, myelomalacia or direct involvement of the meninges and cord by tubercular infection and inflammation, infective thrombosis, or endarteritis of spinal vessels may also lead to neural loss. Atypical presentations with an absence of disc involvement, localization to a single vertebra and the exclusive involvement of the posterior part of the bone are rare [40]. Subjects with SD due to *Brucella* spp. typically present with back pain accompanied by fever, malaise and weight loss [28]. Diagnosis of SD due to *Brucella* spp. is very difficult, and delay may lead to the rapid progression of disease.

Recently, it has been demonstrated that patients who developed complications had a significant delay in diagnosis compared with those that did not [41].

## 6. Laboratory and Radiologic Findings

In most cases that are due to pyogenic bacteria, laboratory findings are unremarkable, showing only a slight to moderate increase in markers of inflammation. In the study carried out by Fernandez *et al.*, it was evidenced that the mean white blood cell (WBC) count was 10,900/mm^3^ in D cases and 12,600/mm^3^ in vertebral osteomyelitis cases, with several patients with values within the normal range [3]. The mean erythrocyte sedimentation rate (ESR) was 42 mm/hour and 45.6 mm/hour in the same groups, respectively. Even lower values for these markers were reported by Kayser *et al.* [4]. However, the highest values are usually found in younger patients with severe diseases with multiple infectious foci and sepsis and in older children with severe osteomyelitis involving more than one vertebra. Additionally, C-reactive protein (CRP) was found to be normal or only slightly elevated, with the highest values in severe cases [42]. Regarding procalcitonin (PCT), no data are available in children. In adults, this marker seems significantly more reliable than WBC, ESR, or CRP in the identification of pyogenic D and SD. It was reported that the elevation of the PCT concentration is almost always demonstrated in pyogenic D and SD, and this can be useful in the differentiation of these cases from tubercular cases in which these markers can have values in the normal range or only slightly elevated [43].

Regarding imaging, abnormalities may be seen on radiographic film 2–3 weeks into the illness, namely narrowing of the intervertebral disc space and variable degrees of destruction of adjacent vertebral endplates. In some cases, spine radiograph remains within normal limits even after prolonged diseases. Scheuerman *et al.*, who studied a group of 52 patients with D, reported that spinal radiograph was diagnostic in only one patient [29]. However, untreated cases show osteolysis and in a subsequent phase deformity of various entity and bone ankyloses [3,4,29].

Bone scintigraphy with technetium (Tc-99m) can add interesting information [44,45]. Spots of increased radiotracer accumulation highlight inflammatory changes. Unfortunately, degenerative changes can cause false-positive findings. Positron emission tomography with 18 fluorodeoxyglucose (FDG-PET) permits to distinguish infections from degenerative changes in the spine [46]. However, the most sensitive imaging method is MRI, which is currently considered the method of choice for the diagnosis of D and SD. Moreover, MRI provides sufficient details to guide the need for invasive diagnostic procedures. MRI sensitivity is approximately 96%, specificity 93%, and accuracy 94% in contrast to plain radiograph, which was found to have a sensitivity of 82%, specificity of 57% and accuracy of 73% [47]. D and SD can be diagnosed in presence of reduced disc height, disc hypointensity on T1-weighted MRI, disc hyperintensity on T2-weighted MRI images, or disc enhancement. Several MRI patterns and signal intensity alterations have been described to be indicative of spinal infection, including those that are decreased [48,49,50,51]. However, Ledermann *et al.* have demonstrated that hypointensity of the disc on T1-weighted MRI and decreased height of the intervertebral space are considered criteria with low sensitivity and limited clinical use [10]. In atypical manifestations of D and SD, typical signal intensity alterations at MRI may not be detected.

Invasive investigations such as needles aspirations or biopsies are reserved for children who fail to improve with antibiotic therapy, when the presence of atypical microorganisms is suspected, or finally when the lesion of vertebral osteomyelitis mimics a tumoral lesion [1,5]. Samples obtained through these investigations may be tested for pyogenic and non-pyogenic bacteria with culture and if feasible also using PCR (*i.e*., that may increase diagnostic sensitivity).

## 7. Treatment

Contrary to adults, for whom official guidelines have been prepared [52], no guideline for the treatment of pediatric D and SD is available. Some authors question the need for antimicrobial therapy for all the cases of primary D because self-limiting D that resolves without drugs has been observed [53,54,55]. When antibiotic therapy is required due to the severity of clinical presentation, the choice of the drug(s) should be based on the sensitivity tests of the causative agent (if available) or the known sensitivity to antibiotics of the pathogens that commonly cause D and SD in the area where the disease is diagnosed. In general, initially while awaiting laboratory tests, a combination of broad-spectrum antibiotics, including a drug active against *S. aureus*, is given intravenously for 3–4 days [52,53,54,55]. When laboratory tests become available, antibiotics can be changed and therapy simplified according to the results. In case of PCR on throat swabs positive for *K. kingae*, β-lactam antibiotics can be prescribed [24].

Recommendations regarding the duration of treatment vary [56,57,58]. However, intravenous administration is usually continued for several days (generally for two weeks), and if the evolution is favorable, then they are substituted with oral therapy that is maintained for several weeks. Kayser *et al.* used a combination of oxacillin and ampicillin for at least 14 days followed by lincomycin until the pain was reduced and inflammation markers were normalized [4]. In most cases, Fernandez *et al.* used a semisynthetic penicillin, usually nafcillin, for intravenous therapy and dicloxacillin or a first-generation cephalosporin for oral therapy [3]. The duration ranged from 10 days to 3–4 weeks. However, considering the increase in the incidence of methicillin-resistant *S. aureus* (MRSA) in both the hospital and the community [59], it is reasonable to think that the present initial therapy of D and SD, particularly in infants and in older children with severe clinical manifestations, is based on different antibiotics, including vancomycin in adequate dosage [60] or other antibiotics active against MRSA [61].

Together with antibiotic therapy and as monotherapy when the administration of antimicrobial drugs is deemed not necessary, bed rest allows the infection to heal and maintains the spine in a normal position to prevent even worse deformities from occurring [4,54,62]. Cervical spine involvement may require traction. The failure of conservative measures or the presence of complications at diagnosis demands surgery. Clinical conditions that require surgery are compression of neural elements, mechanical derangement (*i.e*., instability, malalignment, severe bone destruction) and intractable pain [62,63,64]. However, staging, surgical access, and usage of instrumentation or cages are not defined. Moreover, minimally invasive and endoscopic surgery, which have been largely performed in adults with good results [65], is not usually performed in children, particularly in the youngest.

In cases due to *M. tuberculosis*, therapy with four antituberculosis drugs (*i.e.*, isoniazid, rifampicin, pyrazinamide, and ethambutol) at standard dosages should be started promptly pending microbiological results [66]. If the organism is susceptible, then ethambutol and pyrazinamide can be stopped after 8 weeks, whereas the remaining drugs must be continued to ensure a global therapy of 9–12 months [37,66]. Problems may arise where there are high rates of *M. tuberculosis* resistance to first-line drugs [67]. In this case, second-line drugs must be prescribed, taking into account their penetration into skeletal tissue. Surgery is reserved for cold abscesses and for cases with neurologic problems not modified by the first 2–3 months of drug therapy.

Regarding *Brucella* spp. spinal infections, currently the most commonly used antibiotics are tetracycline, rifampicin, aminoglycosides, trimethoprim-sulfamethoxazole, and quinolones [68]. In children, considering the problems related to the use of quinolones and tetracycline, a combination of trimethoprim-sulfamethoxazole is recommended for approximately 3–6 months [69].

## 8. Conclusions

Despite being rare, infectious discitis (D) and infectious spondylodiscitis (SD) are important diseases in children. In most cases, the diseases are mild and can resolve spontaneously or with simple immobilization of the spine. However, in some cases, they can have a very negative evolution. Diagnosis of D and SD and differentiation of mild from severe cases can be difficult because initial signs and symptoms are nonspecific, and little advantage is derived from the use of laboratory tests and conventional radiography.

D and SD must be considered in all cases where a child suffers from back pain, when movements are reduced, or when an unexplained irritability is manifested. When all of the more common reasons for these signs and symptoms can be excluded, then an MRI can be useful for the diagnosis, reducing the risk of bone lesions requiring surgical interventions and/or the development of a permanent alteration of spine mobility. Particular attention must be paid to the identification of the causative infectious agent and its sensitivity to antibiotics, remembering that traditional culture frequently leads to negative results and modern molecular methods can significantly increase the detection rate. Several different bacterial pathogens can be the cause of D and SD, and in some cases, particularly those due to *S. aureus*, *K. kingae*, *M. tuberculosis* and *Brucella* spp., the appropriate choice of drug for the infecting pathogens is critical to achieve cure.

## Figures and Tables

**Table 1:** Main etiologic agents for discitis (D) and spondylodiscitis (SD) in pediatric age and their characteristics.

Pathogen	Characteristics
*Staphylococcus aureus*	Involved in approximately 80% of the cases that occur in the first months of life and in older children
*Kingella kingae*	Main pathogens in children between 6 months and 4 years
Coagulase-negative *Staphylococcus*, α-hemolytic *Streptococcus*, *Streptococcus* *pneumoniae*, and Gram-negative rods such as *Escherichia coli* and *Salmonella* spp.	Less frequently identified
*Mycobacterium tuberculosis*	Mainly diagnosed in some developing or emerging countries, but reported also in industrialized countries
*Brucella* spp.	Unpasteurized goat cheese consumption
Fungi (*i.e.*, *Aspergillus* spp., *Candida* spp. and *Cryptococcus neoformans*)	Mainly reported in immunocompromised patients
